# Risk factors for suboptimal glycemic control in pediatrics with type 1 diabetes mellitus: a cross-sectional study

**DOI:** 10.1038/s41598-024-57205-9

**Published:** 2024-03-29

**Authors:** Mobin Ghazaiean, Behnam Najafi, Daniel Zamanfar, Mohammad Javad Alipour

**Affiliations:** 1 Student Research Committee, Faculty of Medicine, Mazandaran University of Medical Sciences, Sari, Iran; 2 Gut and Liver Research Center, Non-Communicable Disease Institute, Mazandaran University of Medical Sciences, Sari, Iran; 3 Gastrointestinal Research Center, Non-Communicable Disease Institute, Mazandaran University of Medical Sciences, Sari, Iran; 4 Department of Pediatric Endocrinology, Diabetes Research Center of Mazandaran, Mazandaran University of Medical Sciences, Sari, Iran

**Keywords:** Type 1 diabetes, Pediatric, Glycemic control, Hemoglobin A1c, Endocrinology, Risk factors

## Abstract

The objective of this research is to analyze the influence of various factors on glycemic control in pediatrics with type 1 diabetes mellitus (T1DM). The study, a cross-sectional analysis, involved 221 T1DM patients below 18 years old who visited our clinic between 2011 and 2020, predating the COVID-19 outbreak. Out of the initial pool, 204 participants were chosen based on specific criteria. By computing odds ratios and 95% confidence intervals, we determined the correlation between these factors and achieving optimal glycemic control (HbA1c < 7.5%). Of the 204 individuals, 55.9% (113 patients) were female. The average age at diagnosis was 6.93 ± 3.9 years. Mean HbA1c (A1C) level of optimal and suboptimal groups were 6.97, 95% CI 6.84 to 7.1 and 8.86, 95% CI 8.68 to 9.03, respectively (p-value < 0.001). Fifty patients had optimal glycemic control and 154 people experienced suboptimal glycemic control during the follow-up that the prevalence of each of them was 24.51, 95% CI 18.7 to 31 and 75.49, 95% CI 68.99 to 81.22, respectively. In the assessment of risk factors associated with suboptimal glycemic control, patients aged 10–14 years had the highest likelihood of experiencing suboptimal glycemic control (crude odds ratio [COR] 3.12, 95% CI 1.04 to 9.3), followed by duration of diabetes (COR 2.85, 95% CI 1.2 to 6.8), which both were significant. By utilizing multivariable logistic regression analysis, a noteworthy finding emerged. It was revealed that patients aged 10–14 years exhibited a significant association with suboptimal glycemic control, [adjusted odds ratio (AOR) 4.85, 95% CI 1.32 to 17.7]. Additionally, a statistically significant correlation was identified between individuals with a body mass index (BMI) falling within the ≥ 95th percentile category and suboptimal glycemic control, Cramer’s V = 0.21, p-value = 0.01. Our research has revealed a significant correlation between patients aged 10–14 years and obese individuals (BMI ≥ 95th) with suboptimal glycemic control. It is crucial to consider these factors as they can offer valuable insights during diagnosis, highlighting the increased risk of long-term suboptimal glycemic control.

## Introduction

Type 1 diabetes mellitus (T1DM) stands as the second most prevalent chronic condition affecting children, marked by insulin deficiency and persistent hyperglycemia. The imperative for the sustained well-being of these individuals lies in continuous care and treatment^1^. Over the recent years, the global incidence of T1DM has seen a steady annual increase of 3 to 5%^2^, with China exhibiting the lowest rates and Finland the highest. Nevertheless, this pattern is now showing signs of evolution^3,4^.

The American Diabetes Association (ADA) recommends maintaining a target A1C level of less than 7.5% for optimal glycemic control in children and adolescents with T1DM who have limited access to analog insulin, advanced insulin delivery technology, and continuous glucose monitoring methods^5^. However, achieving this target is crucial in preventing both long-term complications like retinopathy and acute, life-threatening issues such as diabetic ketoacidosis^6,7^. Prolonged exposure to high glucose levels in young individuals can lead to decreased insulin sensitivity and behavioral changes, increasing the risk of complications over time^8^. Recent researches have uncovered a significant link between demographic factors, including age, gender, BMI, family history of diabetes, insulin dosage, frequency of self-monitoring, type of insulin therapy, and adherence to treatment, with glycemic control^9,10^. Factors influencing glycemic control in pediatric populations have been extensively studied, yet a comprehensive understanding of the specific determinants remains elusive^9,11^. Research on this topic in Iran is scarce, but prevailing evidence indicates that a significant proportion of Iranian children with T1DM struggle to achieve optimal glycemic control^12^.

Despite extensive research on the effects of various factors on glycemic control in T1DM^9,11^, this study marks the first comprehensive investigation into the factors influencing glycemic control in individuals with T1DM in northern Iran. Understanding the determinants of A1C levels can significantly enhance the delivery of effective long-term treatment and care for patients while also aiding in the prevention of both acute and chronic complications associated with T1DM. Regrettably, healthcare providers currently lack sufficient tools to identify patients at high risk of deteriorating glycemic control, making it challenging to provide preventive care and prevent a worsening of diabetes management. This study aimed to present data on the prevalence of glycemic control and the influencing factors in individuals with T1DM.

## Methods

### Ethics

All participants and their families were fully briefed on the study's conditions. It was emphasized during the counseling session that they had the option to withdraw from the study at any time. Additionally, participants and their families were assured that their involvement was voluntary and would not impact the treatment and care provided to the patients. The significance of active collaboration to complete the checklist successfully, meet research objectives, and ensure the accuracy of patient information was underscored. All project participants were guaranteed the confidentiality of their information, with all researchers strictly adhering to this pledge. The research was conducted in compliance with applicable guidelines and regulations, with informed written consent obtained from all participants or their legal guardians prior to their involvement. Despite the nature of this study being a cross-sectional analysis of routinely collected patient data, written informed consent was still diligently collected. The ethics committee at Mazandaran University of Medical Sciences has verified that the study upholds the ethical principles outlined in the Declaration of Helsinki (1964) and its subsequent revisions, as well as other pertinent ethical standards (the ethical code: IR.MAZUMS.RIB.REC.1402.14495).

### Study design and population

In a comprehensive investigation, this cross-sectional study delved into the influence of demographic data, disease-related information, and epidemiological factors on glycemic control among 221 children and adolescents with T1DM treated at the Bou Ali Sina Hospital diabetes center between 2011 and 2020. The pediatric diabetes clinic at our facility stands as the primary referral center for T1DM patients in Mazandaran province, drawing the majority of its patients from the region's urban and rural areas. This study adopted a census sampling approach, encompassing all children and adolescents diagnosed with T1DM over the span of a decade. Inclusion criteria encompassed individuals aged 18 or younger with T1DM. Exclusion criteria included maturity-onset diabetes of the young (MODY), lack of patient or guardian consent, and medical record deficiencies. A total of 204 patients met the specified criteria for study inclusion. Importantly, all participants received their diagnoses before the emergence of the COVID-19 pandemic.

The 2024 ADA Standards of Care suggest that achieving optimal glycemic control for individuals under 18 years old, who lack access to analog insulin, advanced insulin delivery technology, and continuous glucose monitoring methods, could be considered less stringent with an A1C target of less than 7.5%^5^. This study categorized T1DM patients into two groups: Group 1 maintained an A1C below 7.5% during the follow-up period, while Group 2 had an average A1C level of 7.5% or higher.

We opted to analyze a 10-year timeframe to encompass the broad spectrum of potential factors impacting glycemic control. Furthermore, we endeavored to leverage possible confounders to adjust the impact of these factors on glycemic control in T1DM patients. This approach enables us to achieve a thorough and precise comprehension of their influence. We have pinpointed potential confounding factors that we believe could impact the outcome of our study. These factors encompass the serum levels of zinc, magnesium, and vitamin D, which were deliberately incorporated into the study's design. Our selection of these minerals was informed by a thorough literature review and physiological rationale. Previous evidence suggests a correlation between minerals like magnesium, zinc, and vitamin D and A1C% levels. In consideration of the impact of these factors on glycemic control^13,14^, the average levels of zinc, magnesium, and vitamin D remained consistent across both groups throughout the follow-up period to serve as potential confounding variables for calculating the adjusted odds ratio. The mean serum levels of zinc, magnesium, and vitamin D in optimal and suboptimal groups were 92.63 ± 13.3 vs. 89.59 ± 14.4, 2.06 ± 0.2 vs. 1.96 ± 0.2, and 30.02 ± 13 vs. 29.31 ± 25, respectively. In addition, we identified a set of potential confounders during the study. These were selected based on disparities between variables in the two groups, including age of diabetes onset, duration of diabetes, BMI, and season of diabetes onset as shown in Table 1. To assess the impact of these confounders individually, we incorporated them into various models. Ultimately, in the final model, we integrated all factors to precisely determine their collective effect on the outcome sizes.

**Table 1:** Basic characteristics, epidemiological and disease-related information of T1DM patients.

Characteristics of patients	Total	Group 1	Group 2	P-value
		HbA1c < 7.5%	HbA1c ≥ 7.5%	
		N = 50 (24.51%)	N = 154 (75.49%)	
Age at diagnosis, year (mean ± SD)	6.93 ± 3.9	6.17 ± 3.9	7.18 ± 3.9	0.1
Age subcategories, years (%)				0.15
1 ≤ age < 4.99	64 (31.6)	18 (36.7)	46(30)	
5–9.99	70 (34.6)	20 (40.8)	50(32.6)	
10–14.99	44 (21.7)	5 (10.2)	39(25.4)	
15–17.99	24 (11.8)	6 (12.2)	18(11.7)	
Gender (%)				0.75
Female	113 (55.9)	27 (54)	86(56.5)	
Male	89 (44)	23 (46)	66(43.4)	
Duration of diabetes, years (%)				**0.04**
1	41 (20)	16(32)	25(16.2)	
1 < duration ≤ 5	82 (40.1)	15(30)	67(43.5)	
> 5	81 (39.7)	19(38)	62(40.2)	
Birth weight (g, mean ± SD)	3249.3 ± 507	3205.3 ± 621	3263.5 ± 466	0.49
Body mass index(BMI, %)				0.08
BMI < 5th	30 (17.1)	8(18.6)	22(16.6)	
5th ≤ BMI < 85th	110 (62.8)	29(67.4)	81(61.3)	
85th ≤ BMI < 95th	18 (10.2)	6(13.9)	12(9)	
BMI ≥ 95th	17 (9.7)	0	17(12.8)	
Season (%)				0.34
Autumn	52 (28.1)	16 (34.7)	36 (25.9)	
Winter	54 (29.1)	14 (30.4)	40 (28.7)	
Spring	41 (22.1)	6 (13)	35 (25.1)	
Summer	38 (20.5)	10 (21.7)	28 (20.1)	
Order of birth (%)				0.46
First	114 (56.1)	26(53)	88 (57.1)	
Second	76 (37.4)	19(38.7)	57 (37)	
Third	9 (4.4)	3(6.1)	6 (3.9)	
Fourth	2 (0.98)	0	2 (1.3)	
Fifth	1 (0.49)	1(2)	0	
Sixth	1 (0.49)	0	1 (0.65)	
Blood group (%)				0.83
O positive	66 (35.8)	16 (36.3)	50 (35.7)	
O negative	11 (5.9)	3 (6.8)	8 (5.7)	
A positive	44 (23.9)	10 (22.7)	34 (24.2)	
A negative	5 (2.7)	2 (4.5)	3 (2.1)	
B positive	40 (21.7)	9 (20.4)	31 (22.1)	
B negative	5 (2.7)	0	5 (3.5)	
AB positive	13 (7)	4(9)	9 (6.4)	
Location (%)				0.19
City	57 (55.3)	42 (84)	15 (75.1)	
Village	46 (44.6)	8 (16)	38 (24.8)	
Parental ratio (%)				0.25
Consanguineous	40 (19.6)	7 (14)	33 (21.4)	
Non-consanguineous	164 (80.3)	43 (86)	121 (78.5)	
Type of delivery (%)				0.66
Natural vaginal delivery	66 (0.32)	15 (30)	51 (33.3)	
Cesarean	137 (0.67)	35 (70)	102 (66.6)	
Type of birth (%)				**0.049**
Term	185 (93.4)	41(87.2)	144 (95.3)	
Pre-term	13 (6.5)	6 (12.7)	7 (4.6)	
Duration of breastfeeding, months (%)				0.17
< 6	27 (13.7)	10 (21.2)	17 (11.3)	
6–12	6(2.7)	2 (4.2)	4 (2.6)	
12–18	13 (6.6)	1 (2.1)	12 (8)	
18–24	151 (77)	34 (72.3)	117 (78)	
Age of supplementary feeding, months (%)				0.19
< 6	8 (4)	0	8(5.3)	
6	186 (94.8)	46 (97.8)	140 (93.9)	
> 6	2(1.2)	1 (2.1)	1 (0.67)	
Family history of diabetes (%)				0.92
Positive	76 (37.4)	19 (38)	57 (37.2)	
Negative	127 (62.5)	31 (62)	96 (62.7)	
Family history of dyslipidemia (%)				0.35
Positive	63 (31.3)	18 (36.7)	45 (29.6)	
Negative	138 (67.9)	31 (63.2)	107 (70.3)	
Family history of autoimmune disease (%)				0.22
Hypothyroidism	39 (19.2)	6 (12)	33 (21.5)	
Graves	5 (2.4)	2 (4)	3 (1.9)	
Multiple sclerosis	1 (0.49)	1 (2)	0	
Rheumatism	4 (1.97)	1 (2)	3 (1.9)	
Negative	154 (75.8)	40 (80)	114 (74.5)	
Past medical history (%)				0.07
Celiac	6 (2.9)	3(6)	3(1.97)	
Hashimoto	27 (13.3)	3(6)	24(15.7)	
Celiac & Hashimoto	4 (1.9)	2(4)	2(1.3)	
Down syndrome & Hashimoto	1 (0.49)	1(2)	0	
Hypothyroidism	2 (0.99)	1(2)	1(0.66)	
Allergy	2 (0.99)	1(2)	1(0.66)	
Favism	3 (1.4)	2(4)	1(0.66)	
Dyslipidemia	6 (2.9)	2(4)	4(2.63)	
Favism & dyslipidemia	1 (0.49)	1(2)	0	
Asthma	1 (0.49)	1(2)	0	
Hashimoto & dyslipidemia	3 (1.4)	1(2)	2(1.32)	
Lymphoma	1 (0.49)	1(2)	0	
Down syndrome & celiac & hypothyroidism	1 (0.49)	0	1(0.66)	
Dystrophy	1 (0.49)	0	1(0.66)	
Seizure & asthma	1 (0.49)	0	1(0.66)	
Graves	1 (0.49)	0	1(0.66)	
Negative	141 (69.8)	31(62)	110(72.3)	
DKA at diagnosis				0.87
Positive	75 (36)	18 (36)	57 (37.2)	
Negative	128 (63)	32 (64)	96 (62.7)	
C-peptide (ng/ml)	0.47 ± 0.5	0.52 ± 0.38	0.46 ± 0.54	0.11
Anti-TPO				0.25
Positive	35 (17.3)	6 (12)	29 (19)	
Negative	167 (82.6)	44 (88)	123 (80.9)	
Anti-TTG				0.10
Positive	11 (5.4)	5(10)	6(3.9)	
Negative	192 (94.5)	45(90)	147(96)	
Pancreas auto-antibody profile				0.69
Positive	143 (76)	34(73.9)	109(76.7)	
Negative	45 (23.9)	12(26)	33(23.2)	
Anti-GAD				0.39
Positive	104 (58.1)	22(52.3)	82(59.8)	
Negative	75 (41.8)	20(47.6)	55(40.1)	
Anti-ICA				0.87
Positive	115 (61.8)	28(60.8)	87(62.1)	
Negative	71 (38.1)	18(39.1)	53(37.7)	
Anti-IAA				0.82
Positive	47 (25.4)	12(26.6)	35(25)	
Negative	138 (74.5)	33(73.3)	105(75)	
Anti-IA2				0.91
Positive	85 (45.9)	21(46.6)	64(45.7)	
Negative	100 (54)	24(53.3)	76(54.2)	
Mean TC (mg/dl)	164.2 ± 28.1	157.8 ± 31.2	166.3 ± 26.7	0.10
Mean LDL (mg/dl)	87 ± 21.9	82 ± 21.4	88.6 ± 21.8	0.059
Mean HDL (mg/dl)	51.4 ± 10	50.2 ± 10.3	51.7 ± 9.9	0.30
Mean TG (mg/dl)	94.3 ± 44.9	84.1 ± 26.6	97.7 ± 49	**0.04**
Treatment regimens (%)				0.8
NPH & regular	139 (68.8)	35(70)	104(68.4)	
Lantus & novorapid	47 (23.2)	12(24)	35(23)	
Lantus & apidra	7 (3.4)	2(4)	5(3.2)	
Levemir & apidra	9 (4.4)	1(2)	8(5.2)	

Data are shown as standard deviation ± mean or (percentage) number. Bold values indicate significant changes between the groups.

*P* value was obtained by Fisher’s exact test and Student *t* test.

*TC* total cholesterol, *LDL* low-density lipoprotein, *HDL* high-density lipoprotein, *TG* triglycerides.

### Data collection

During face-to-face interviews and medical record reviews, a comprehensive checklist was utilized to gather essential data by two physicians including demographic details like age at diagnosis, sex, birth weight (gram), body mass index (BMI, kg/m^2^), duration of diabetes, season of diabetes onset, order of birth, blood group (A, B, AB, O), location (city or village), parental ratio (consanguineous or non-consanguineous), type of delivery (natural vaginal delivery or caesarean section), type of birth (term or pre-term), duration of breastfeeding, age of supplementary feeding, family history of diabetes, family history of dyslipidemia, family history of autoimmune diseases (hypothyroidism, graves, multiple sclerosis and rheumatism), past medical history (PMH; celiac, hashimoto, hypothyroidism, down syndrome, allergy, favism, dyslipidemia, asthma, lymphoma, dystrophy, seizure and graves), initial presentation of patients (with or without DKA), c-peptide level, anti-TPO, anti-TTG, pancreas auto-antibody profile, anti-GAD, anti-ICA, anti-IAA and anti-IA2, serum lipid profile including total cholesterol, low-density lipoprotein (LDL), high-density lipoprotein (HDL), triglycerides (TG), serum zinc level, serum magnesium level, serum vitamin D level, treatment regimens (NPH & Regular, Lantus & Novorapid, Lantus & Apidra, and Levemir & Apidra).

### Anthropometric evaluation

The World Health Organization (WHO) growth curves are a valuable tool for determining a patient's BMI percentile. This percentile is calculated using the patient's age, gender, and weight in kilograms divided by squared height in meters. The results can then be classified into four categories: underweight (BMI < 5th), normal weight (5th ≤ BMI < 85th), overweight (85th ≤ BMI < 95th), and obese (BMI ≥ 95th)^16^.

### Outcomes

The main aim of this study is to analyze and compare the demographic characteristics, epidemiological data, clinical information, and laboratory results of the two groups. Additionally, we investigated the relationship between these factors and achieving optimal glycemic control.

### Statistical analysis

The racked data were analyzed with STATA software version 17 (StataCorp, College Station, TX). Number (percent) or mean standard deviation (SD) was used to present the data. We used a chi-square or two-sample t-test (Mann–Whitney U for numeric data samples which were not distributed normaly) to compare dichotomous or numeric data between the defined groups as per cut-off point for A1C (7.5%). Before using the parametric test, we ensured that the data followed a normal distribution through the Shapiro–Wilk test and histogram. By applying logistic regression and estimating odds ratios [ORs, with a 95% confidence interval (CI)], we assessed potential risk factors for poor performance in keeping a state of optimal glycemic control as A1C less than 7.5%, respectively. Several adjusted models were set to fix the potential effects of some potential confounders based on Table 1 results and previously valid data, including age of diabetes onset, duration of diabetes, BMI, season of diabetes onset, zinc (mg/dL), magnesium (mg/dL) and 25-OHD3 level (ng/mL) levels. The model’s goodness of fit was evaluated using the ESTAT GOF command upon running each model. Across estimating a VIF index (variance inflation factor), multicollinearity between the independent variables was also tested in the final model using the LMCOL command. All calculated VIF values were less than 2.5. A P-value under 0.05 was seen as the statistical significance level.

### Consent to participate

Informed consent was obtained from all subjects and/or their legal guardian(s) as minor/illiterate participants are involved.

## Result

Out of the 204 participants in the study, 50 were assigned to group one with A1C levels below 7.5%, while the remaining 154 were placed in group two. Among the participants, 55.9% (113) were female. The average age of the patients was 6.93 ± 3.9 years. The mean A1C levels for the optimal and suboptimal groups were 6.97 (95% CI 6.84 to 7.1) and 8.86 (95% CI 8.68 to 9.03) respectively (p-value < 0.001). The prevalence of optimal and suboptimal glycemic control was 24.51% (95% CI 18.7 to 31) and 75.49% (95% CI 68.99 to 81.22) respectively. A comparison between the two groups indicated significant differences in diabetes duration, type of birth (term or pre-term), and mean TG level. A summary of the participants’ findings is mentioned in Table 1.

The risk assessment of factors associated with glycemic control revealed that those patients aged 10–14 years had the highest likelihood of suboptimal glycemic control (COR 3.12, 95% CI 1.04 to 9.3). Additionally, individuals with a diabetes duration of 1–5 years had a COR of 2.85, with a 95% CI of 1.2 to 6.8. Notably, the subgroup of patients with a BMI ≥ 95th percentile showed a significant association with suboptimal glycemic control (p-value = 0.01). For detailed information on the calculated CORs of these factors, please refer to Table 2.

**Table 2:** Suboptimal glycemic control risk assessment of potential factors.

Variables	Model 1	Model 2	Model 3	Model 4
Age subcategories, years (%)				
5–9.99	1	1	1	1
< 5	1.02, 0.48 to 2.17	1.11, 0.49 to 2.48	1.12, 0.45 to 2.75	1.18, 0.44 to 3.18
10–14.99	**3.12, 1.04 to 9.3**	**3.23, 1.08 to 9.66**	**4.22, 1.21 to 14.65**	**4.85, 1.32 to 17.7**
15–17.99	1.2, 0.41 to 3.48	0.42, 0.025 to7.22	**8.83, 1.01 to 76.86**	–
Gender				
Male	1	1	1	1
Female	1.10, 0.58 to 2.11	0.95, 0.46 to 1.97	1.27, 0.57 to 2.8	1.05, 0.44 to 2.55
Duration of diabetes, years				
1	1	1	1	1
1 < duration ≤ 5	**2.85, 1.2 to 6.8**	2.24, 0.91 to 5.51	2.16, 0.76 to 6.08	1.95, 0.63 to 6.00
> 5	2.08, 0.91 to 4.76	1.46, 0.57 to 3.71	1.88, 0.66 to 5.29	1.13, 0.35 to 3.67
Body mass index				
5th ≤ BMI < 85th	1	1	1	1
BMI < 5th	0.98, 0.39 to 2.46	1.18, 0.45 to 3.14	0.80, 0.29 to 2.20	0.91, 0.30 to 2.72
85th ≤ BMI < 95th	0.71, 0.24 to 2.09	0.66, 0.21 to 2.06	0.57, 0.16 to 2.03	0.77, 0.20 to 2.90
BMI ≥ 95th	–	–	–	–
Disease onset season				
Autumn	1	1	1	1
Winter	1.26, 0.54 to 2.97	1.15, 0.45 to 2.97	1.11, 0.4 to 3.11	0.83, 0.26 to 2.61
Spring	3.27, 0.88 to 7.57	**3.28, 1.06 to 10.17**	1.79, 0.56 to 5.65	2.37, 0.66 to 8.50
Summer	1.24, 0.48 to 3.17	1.26, 0.48 to 3.3	1.04, 0.33 to 3.26	1.09, 0.31 to 3.83
Order of birth				
First	1	1	1	1
Second	0.88, 0.44 to 1.75	0.89, 0.41 to 1.93	0.83, 0.37 to 1.89	1.09, 0.43 to 2.78
Third	0.59, 0.13 to 2.55	0.91, 0.16 to 5.1	0.51, 0.096 to 2.78	1.59, 0.22 to 11.2
Blood group				
O positive	1	1	1	1
O negative	0.85, 0.2 to 3.6	0.92, 0.19 to 4.30	0.26, 0.04 to 1.56	0.24, 0.03 to 1.72
A positive	1.08, 0.44 to 2.69	1.17, 0.42 to 3.24	1.64, 0.56 to 4.78	2.40, 0.69 to 8.27
A negative	0.48, 0.07 to 3.2	1.10, 0.10 to 12	–	–
B positive	1.1, 0.43 to 2.8	1.12, 0.4 to 3.14	1.34, 0.45 to 3.95	1.11, 0.33 to 3.70
AB positive	0.72, 0.19 to 2.68	1.44, 0.25 to 8.2	2.02, 0.21 to 19.06	2.30, 0.21 to 25.06
Location				
Village	1	1	1	1
City	0.57, 0.24 to 1.34	0.66, 0.26 to 1.68	0.43, 0.14 to 1.25	0.72, 0.23 to 2.24
Parental ratio				
Non-consanguineous	1	1	1	1
Consanguineous	1.67, 0.68 to 4.08	1.48, 0.57 to 3.82	2.02, 0.67 to 6.02	1.73, 0.53 to 5.62
Type of delivery				
Normal vaginal delivery	1	1	1	1
Cesarean	0.85, 0.42 to 1.71	0.76, 0.34 to 1.68	0.85, 0.37 to 1.96	0.78, 0.30 to 2.01
Mode of delivery				
Term	1	1	1	1
Pre-term	0.33, 0.1 to 1.05	0.36, 0.10 to 1.33	0.28, 0.073 to 1.09	0.31, 0.07 to 1.41
Family history of diabetes				
Negative	1	1	1	1
Positive	0.96, 0.5 to 1.87	0.85, 0.39 to 1.82	1.01, 0.46 to 2.25	0.82, 0.33 to 2.02
Family history of dyslipidemia				
Negative	1	1	1	1
Positive	0.72, 0.36 to 1.43	0.64, 0.29 to 1.4	0.79, 0.33 to 1.87	0.51, 0.19 to 1.38
Family history of autoimmune disease				
Negative	1	1	1	1
Hypothyroidism	1.92, 0.74 to 4.98	2.47, 0.85 to 7.22	2.08, 0.65 to 6.67	2.00, 0.56 to 7.15
Graves	0.52, 0.08 to 3.29	0.70, 0.059 to 8.44	–	–
Rheumatism	1.05, 0.1 to 10.4	1.04, 0.096 to 11.2	0.54, 0.04 to 6.81	0.54, 0.037 to 7.81
Duration of breastfeeding, months				
18–24	1	1	1	1
< 6	0.49, 0.2 to 1.18	0.68, 0.25 to 1.81	0.49, 0.15 to 1.59	0.66, 0.17 to 2.53
6–12	0.58, 0.1 to 3.3	0.89, 0.08 to 9.67	0.52, 0.04 to 6.69	0.65, 0.04 to 10.6
12–18	3.48, 0.43 to 28.2	3.00, 0.36 to 24.7	3.25, 0.38 to 27.3	3.78, 0.36 to 39.0
Age of supplementary feeding, months				
6	1	1	1	1
> 6	0.32, 0.02 to 5.42	0.43, 0.02 to 7.66	0.25, 0.014 to 4.32	0.51, 0.02 to 9.58
Hashimoto				
Negative	1	1	1	1
Positive	2.25, 0.67 to 7.45	5.73, 0.72 to 45.3	2.38, 0.50 to 11.2	4.41, 0.5 to 38.56
DKA at diagnosis				
Negative	1	1	1	1
Positive	1.05, 0.54 to 2.05	1.25, 0.57 to 2.70	1.13, 0.49 to 2.61	1.25, 0.48 to 3.25
Anti-TPO				
Negative	1	1	1	1
Positive	1.72, 0.66 to4.46	2.60, 0.72 to 9.36	1.98, 0.62 to 6.27	3.10, 0.63 to 15.12
Anti-TTG				
Negative	1	1	1	1
Positive	0.36, 0.1 to 1.27	0.36, 0.98 to 1.33	0.39, 0.1 to 1.6	0.36, 0.078 to 1.71
Pancreatic auto-antibody				
Negative	1	1	1	1
Positive	1.16, 0.54 to 2.51	1.12, 0.48 to 2.62	0.64, 0.23 to 1.77	0.57, 0.18 to 1.79
Anti-GAD				
Negative	1	1	1	1
Positive	1.35, 0.67 to 2.72	1.03, 0.48 to 2.23	1.04, 0.45 to 2.37	0.72, 0.27 to 1.89
Anti-ICA				
Negative	1	1	1	1
Positive	1.05, 0.53 to 2.09	1.16, 0.54 to 2.47	0.89, 0.39 to 2.03	0.87, 0.34 to 2.2
Anti-IAA				
Negative	1	1	1	1
Positive	0.91, 0.42 to 1.97	1.009, 0.43 to 2.32	0.71, 0.28 to 1.79	0.93, 0.34 to 2.56
Anti-IA2				
Negative	1	1	1	1
Positive	0.96, 0.48 to 1.89	1.28, 0.60 to 2.70	0.84, 0.38 to 1.9	1.14, 0.46 to 2.86
Treatment regimens				
NPH & regular	1	1	1	1
Lantus & novorapid	0.98, 0.45 to 2.1	1.02, 0.36 to 2.88	1.66, 0.6 to 4.56	1.45, 0.42 to 4.99
Lantus & apidra	0.84, 0.15 to 4.55	0.34, 0.05 to 2.29	1.88, 0.19 to 17.8	0.70, 0.05 to 9.74
Levemir & apidra	2.69, 0.32 to 22.6	2.45, 0.28 to 21.48	1.42, 0.15 to 13.0	2.05, 0.20 to 20.3

Model 1: unadjusted odds ratios (95%CI).

Model 2: ORs (95%CI) were adjusted according to age of diabetes onset, duration of diabetes, BMI, and season of diabetes onset.

Model 3: ORs (95%CI) were adjusted according to zinc, magnesium and vitamin D levels.

Model 4: ORs (95%CI) were adjusted according to age of diabetes onset, duration of diabetes, BMI, season of diabetes onset, zinc, magnesium, and vitamin D levels.

Significant values are in bold.

In model 2, after adjusting for potential confounders such as age of diabetes onset, duration of diabetes, BMI, and season of diabetes onset, patients aged 10–14 years exhibited a significant correlation with suboptimal glycemic control, showing an adjusted odds ratio (AOR) of 3.23 with a 95%CI of 1.08 to 9.66. Moreover, individuals diagnosed in the spring had 3.28 times higher odds of suboptimal glycemic control, with a 95% CI of 1.06 to 10.17. Moving to model 3, which accounted for serum levels of zinc, magnesium, and vitamin D as potential confounders, both patients aged 10–14 years and those over 15 years showed a significant association with suboptimal glycemic control, with AORs of 4.22 (95% CI 1.21 to 14.65) and 8.83 (95% CI 1.01 to 76.86), respectively.

In model 4 of the study, the results from multivariate logistic regression analysis revealed that patients aged 10–14 years had the highest adjusted odds of suboptimal glycemic control (AOR 4.85, 95% CI 1.32 to 17.7), even after accounting for all potential confounding factors. Notably, there were no patients with optimal glycemic control in the BMI subcategory of ≥ 95th, indicating a significant correlation with suboptimal glycemic control as shown by Cramer's V (0.21, p-value = 0.01). The adjusted odds ratios of the various factors are mentioned in Table 2.

Cohen’s d was calculated for variables like birth weight and c-peptide level at diagnosis. There was no significant correlation found between birth weight and glycemic control, as indicated by a Cohen's d of -0.11 (95% CI -0.21 to 0.44). Similarly, there was no significant association between c-peptide level at diagnosis and glycemic control, with a Cohen's d of 0.12 (95% CI -0.25 to 0.50).

## Discussion

This research delved into the factors influencing optimal glycemic control in pediatrics with T1DM. It was discovered that patients diagnosed between the ages of 10–14 had the strongest link to poor glycemic control. Additionally, obese patients (BMI ≥ 95th) were notably associated with suboptimal glycemic control. Our study found that 75.4% of patients did not maintain optimal glycemic control during the follow-up period. The challenges of achieving optimal glycemic control in young T1DM patients are evident. An extensive international study involving 44,058 children under 15 with T1DM reported a range of 15.7% to 46.4% achieving optimal glycemic control (A1C cut-off < 7.5%)^18^. In a study of children and adolescents with T1DM in Egypt, it was found that only 45.8% of patients achieved optimal glycemic control^19^. Similarly, a 2019 study of 1095 US children aged 10–17 years with T1DM revealed that just 35.8% had optimal glycemic control (A1C cut-off ≥ 9.5%)^20^. On the other hand, in Spain, 66.6% of 853 T1DM patients under 18 years of age achieved optimal glycemic control^21^. A recent study by Hashemipour et al. in 2021, involving 454 T1DM patients aged 6–18 years, showed that 85.5% had suboptimal glycemic control (A1C cut-off ≥ 7%) ^22^. The fluctuating percentages of optimal glycemic control in studies of children with T1DM may vary due to the diverse cut-off points of A1C across different geographical regions and references. This inconsistency may also stem from differences in demographic characteristics, sample size, living standards, dietary habits, and the presence of structured diabetes education programs^23,24^.

In this study, it was observed that individuals with suboptimal glycemic control tended to be older compared to those with optimal control. Notably, a significant correlation was found between patients aged 10–14 years and suboptimal glycemic control, with odds of 4.8. Previous research has indicated a rise in A1C levels among children and adolescents aged 10–17 years with T1DM^25,26^. Moreover, Kidie et al. noted a 15% higher likelihood of poor glycemic control in individuals under 18 years with increasing mean age^27^. In a cohort study by Clements et al. involving 2218 children and adolescents with T1DM, it was revealed that those diagnosed at an older age (≥ 10 years old) were more prone to experiencing poorer glycemic control^28^. Several studies conducted in Bulgaria, the Amhara region, and Egypt have all found a link between increasing mean age and suboptimal glycemic control^7,19,29^. The potential rationale for the findings of this study may be linked to the fact that the research focuses on a demographic consisting of adolescents, who are particularly vulnerable to inadequate management of their diabetes due to the challenges associated with puberty. Adolescents may struggle to adhere to their prescribed treatment plan effectively. Moreover, the physiological and hormonal changes during puberty, such as increased adipose tissue and insulin resistance, could further contribute to these difficulties^23^. Another contributing factor is the challenge of following a diabetes-focused routine, especially for young children. These difficulties can lead to increased medical complications and suboptimal glycemic control^19,30^. While the current study does not delve into this issue, it is important to note that glycemic control can also be influenced by other disease-specific factors. These include the decline in residual β-cell function as the disease advances, as well as the development of insulin resistance during puberty^31,32^. It is our belief that the decline in glycemic control post-diagnosis cannot be solely attributed to residual β-cell function, commonly referred to as the 'honeymoon phase'. Despite younger children having lower levels of residual β-cell function (up to age 7), they experience a milder deterioration in glycemic control. Additionally, the loss of the 'honeymoon phase' occurs at a similar or even faster pace in younger children^33,34^.

In the present study, we found that obese patients (BMI ≥ 95th percentile) were only present in the group with suboptimal glycemic control. Our results revealed a moderate association between BMI ≥ 95th percentile and the risk of suboptimal glycemic control. This aligns with a cross-sectional study conducted in Austria, Germany, and the United States, which showed a correlation between obesity in children with T1DM and suboptimal glycemic control^35^. However, Hashemipour et al.'s study reported higher A1C values in underweight patients, contrary to our findings^22^. In a 2018 international cross-sectional study, it was found that both underweight and obese patients had a higher rate of suboptimal glycemic control^36^. Subsequent research on T1DM patients aged 6–18 in 2022 further supported this, showing a clear link between abdominal obesity and poorer glycemic control^37^. However, not all studies have confirmed a direct correlation between BMI and A1C levels^38,39^. For instance, a cohort study involving 635 T1DM patients aged 7–24 found no significant difference in A1C levels between underweight, normal weight, and overweight/obese patients. These varying results highlight the complexity of factors influencing glycemic control in these patients^38^. National cohort studies have revealed a concerning trend in adolescents diagnosed with type 1 diabetes, showing an increase in BMI along with higher insulin resistance. The intricate relationship between BMI, daily insulin doses, insulin resistance, severe hypoglycemia, and A1C has garnered attention^40–43^. Despite the typical association of individuals with T1DM being underweight, lifestyle factors such as sedentary habits and a diet rich in high-sugar and high-fat foods have led to weight gain in this group. It is widely recognized that an increase in adipose tissue, especially visceral fat, and a decrease in lean mass can worsen glycemic control^17,44^. In addition to A1C levels, the apolipoproteins (apo) A-I, A-II, and the Apo A-II/Apo A-I ratio are critical factors in the progression of T1DM, influencing both glucose metabolism and the development of cardiovascular complications. Changes in these molecules can hinder glycemic control in individuals with T1DM^45–47^. Increased adipose tissue, especially visceral fat, and decreased skeletal muscle mass are linked to worsened glycemic and lipid metabolism, leading to impaired glycemic regulation and the need for higher insulin doses in T1DM patients^17,44^. The musculature has vital functions in upholding homeostasis and is also interconnected with exocrine actions. The resultant factors are denominated cytokines, myokines, or growth factors, which can fulfill paracrine, autocrine, or endocrine functions. Among the various roles played by these factors, it is feasible to discuss the enhancement in glycemic regulation through the attenuation of insulin resistance and the amelioration of protein and lipid metabolism. Numerous myokines exhibit favorable effects on glucose assimilation and the amelioration of blood glucose levels. Conversely, the pro-inflammatory scenario instigated by the reduction in the secretion of these myokines, which, in conjunction with substandard glycemic control, gives rise to an augmented susceptibility to the development of metabolic syndrome and subsequent cardiovascular complications^46,48,49^.

This study demonstrates that there is no significant correlation between the duration of diabetes and suboptimal glycemic control. This finding is consistent with previous research conducted in Tanzania and India, which also concluded that the duration of diabetes is not a reliable predictor of suboptimal glycemic control^50,51^. In contrast, studies in the Amhara region, Jimma, Shanan Gibe, and Egypt revealed a statistically significant association between the duration of diabetes and suboptimal glycemic control^24,29,52,53^. The potential cause for this occurrence may stem from several factors, including a prolonged duration of the pathological process, reduced insulin production, complications associated with diabetes, and a gradual decline in insulin secretion^54,55^. Additionally, as diabetes mellitus progresses, patients may become less diligent in adhering to their prescribed medication regimen and maintaining optimal glycemic control^29^.

This groundbreaking study delves into the factors influencing glycemic control in individuals with T1DM in northern Iran. By pinpointing potential contributors to suboptimal glycemic management, we shed light on critical aspects for healthcare providers. Understanding the dynamics shaping long-term glycemic control is paramount, particularly as patients’ age and face escalating challenges in maintaining stability. Armed with this knowledge, clinicians can proactively tailor treatment strategies early in the T1DM journey, ensuring personalized and top-tier care for patients.

### Limitations and recommendation for future studies

In light of the cross-sectional design of our study, it is imperative to exercise caution when interpreting the results as they may not accurately portray causal relationships. Therefore, the association should be assessed in prospective studies for a more comprehensive understanding. An additional limitation to consider is the potential for overestimation bias due to the study's cross-sectional nature, necessitating readers to approach the findings with vigilance. Moreover, crucial data on variables such as socioeconomic status, physical activity levels, sedentary behavior patterns, sleep quality, dietary components, blood sugar monitoring frequency, treatment adherence, history of hypoglycemia, and insulin dosage were regrettably unavailable for analysis in relation to the patients. Furthermore, the retrospective design of our study may have led to missing data, potentially impacting the accuracy of our findings. Additionally, the reliance on self-reported information could have introduced recall and desirability biases, further compromising the study's validity. While our diabetes center caters to a significant portion of T1DM patients in the province, it is essential to acknowledge that the study's single-center approach may limit the generalizability of the results to a broader population. The inability to access precise socioeconomic data prior to diagnosis, including parental education and family income, represents a significant constraint. These factors are crucial as they are linked to parental awareness of disease symptoms, timely referrals to medical facilities to avert DKA onset at diagnosis, and ultimately, enhancing long-term glycemic control^15^. Despite dedicated efforts by healthcare providers to optimize insulin therapy during adolescence, there remains a noticeable decline in glycemic control. It is logical to consider that factors influencing adherence to intensified diabetes treatment protocols play a crucial role in the collaborative efforts of patients and providers to mitigate the effects of puberty on insulin needs and glycemic management. It is advisable for forthcoming research to comprehensively investigate all potential mediators impacting glycemic control in young individuals. This additional insight would significantly aid in crafting intervention strategies tailored to their unique developmental stage.

## Conclusion

Our research has revealed a significant link between T1DM patients aged 10–14 years and obesity, leading to suboptimal glycemic control. This finding highlights the critical need to educate families and children in the community on managing T1DM effectively. Understanding these factors is crucial as they can offer valuable insights at the time of diagnosis, particularly regarding the increased risk of poor glycemic control in T1DM patients during ongoing monitoring.

## Data Availability

The datasets used and/or analysed during the current study available from the corresponding author on reasonable request.

## Abbreviations

T1DM: Type 1 diabetes mellitus
COR: Crude odds ratio
BMI: Body mass index
AOR: Adjusted odds ratio
ADA: American Diabetes Association
DKA: Diabetic ketoacidosis
MODY: Maturity-onset diabetes of the young
PMH: Past medical history
TC: Total cholesterol
LDL: Low-density lipoprotein
HDL: High-density lipoprotein
TG: Triglycerides
WHO: World Health Organization
CI: Confidence interval
